# Sirtuin 1-Activating Compounds: Discovery of a Class of Thiazole-Based Derivatives

**DOI:** 10.3390/molecules27196535

**Published:** 2022-10-03

**Authors:** Giulia Bononi, Valentina Citi, Margherita Lapillo, Alma Martelli, Giulio Poli, Tiziano Tuccinardi, Carlotta Granchi, Lara Testai, Vincenzo Calderone, Filippo Minutolo

**Affiliations:** 1 Department of Pharmacy, University of Pisa, Via Bonanno 6, 56126 Pisa, Italy; 2 Center for Instrument Sharing of the University of Pisa (CISUP), Lungarno Pacinotti 43, 56126 Pisa, Italy

**Keywords:** SIRT1, activators, cardioprotection, thiazoles, resveratrol

## Abstract

Sirtuin 1 (SIRT1) is a NAD^+^-dependent deacetylase implicated in various biological and pathological processes, including cancer, diabetes, and cardiovascular diseases. In recent years, SIRT1-activating compounds have been demonstrated to exert cardioprotective effects. Therefore, this enzyme has become a feasible target to treat cardiovascular diseases, and many SIRT1 activators, of a natural or synthetic origin, have been identified. In the present work, we developed thiazole-based SIRT1 activators, which showed remarkably higher SIRT1 activation potencies compared with those of the reference compound resveratrol when tested in enzymatic assays. Thiazole **8**, a representative compound of this series, was also subjected to further pharmacological investigations, where it was proven to reduce myocardial damage induced by an in vivo occlusion/reperfusion event, thus confirming its cardioprotective properties. In addition, the cardioprotective effect of compound **8** was significantly higher than that of resveratrol. Molecular modeling studies suggest the binding mode of these derivatives within SIRT1 in the presence of the p53-AMC peptide. These promising results could pave the way to further expand and optimize this chemical class of new and potent SIRT1 activators as potential cardioprotective agents.

## 1. Introduction

**Figure 1:**
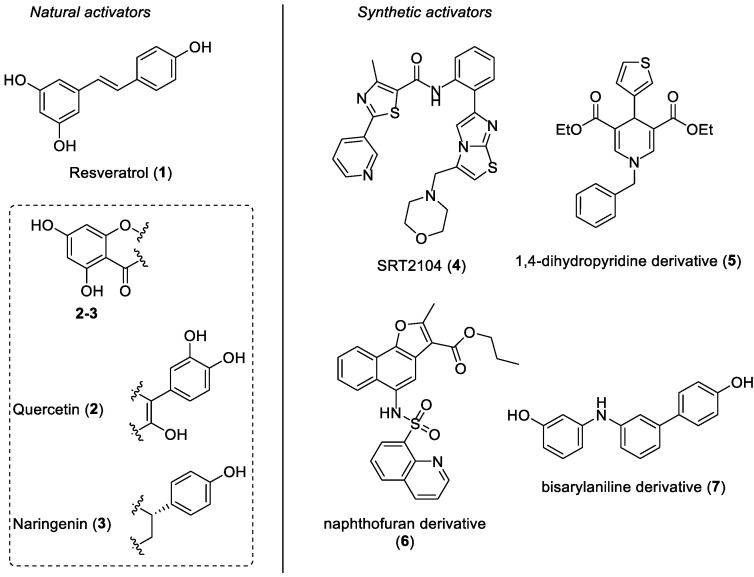
Some representative examples of SIRT1-activating compounds: natural (compounds **1–3**, on the left) and synthetic (compounds **4–7**, on the right) SIRT1 activators.

Sirtuins (SIRTs) are a highly conserved family of nicotinamide adenine dinucleotide (NAD^+^)-dependent enzymes belonging to class III histone deacetylases (HDACs). These proteins catalyze the histone and non-histone deacetylation of lysine residues and their catalytic activity is regulated by NAD^+^ as a cofactor [1]. In mammals, the sirtuin family is composed of seven members (from SIRT1 to SIRT7), which differ from each other in subcellular localization, enzymatic activity, and substrates. SIRTs are implicated in various cellular processes, including metabolic regulation, autophagy, apoptosis, DNA repair, stress resistance, and gene expression [2,3]. SIRT1 is the most studied and the best characterized mammalian sirtuin to date, and it mainly resides in the nucleus, although further studies have also highlighted its presence in the cytosol of some types of cells [4]. This enzyme reversibly deacetylates the ε-acetyl-lysine residues of both histone and non-histone proteins by forming deacetylated targets, together with nicotinamide and 2′-*O*-acetyl-ADP-ribose, in a two-step process: (a) The first step consists of NAD^+^ cleavage together with the covalent attachment of the ADP-ribose unit to the acetyl group of the protein target. (b) In the second step, the hydrolysis of the acetyl-lysine bond generates the 2′-*O*-acetyl-ADP-ribose product [5,6]. SIRT1 plays a central role in the regulation of the cell metabolism, inflammation, longevity, ageing, DNA repair, reactive oxygen species (ROS) production, oxidative stress, and autophagy [7,8,9,10,11]. Therefore, deregulation of SIRT1 functions may induce tissue-specific degenerative processes, thus leading to various human pathologies, including cancer, diabetes, and cardiovascular diseases such as myocardial ischemia/reperfusion injury [2,3,12,13,14,15]. Considering its involvement in the pathophysiology of many human diseases, SIRT1 has become a feasible target to deal with, and many SIRT1-activating compounds have been identified and developed as therapeutic agents in recent years [16,17]. Compounds able to activate SIRT1 can be divided into natural and synthetic activators. Resveratrol (*trans*-3,5,4′-trihydroxystilbene **1**, Figure 1) is a polyphenol extracted from grape skins, red wine, and other edible materials, and it is probably the most relevant SIRT1-activating natural product. This stilbenoid derivative has exerted cardioprotective activities by means of SIRT1 activation in various in vitro and in vivo models of myocardial ischemia/reperfusion injury, and it has been demonstrated to increase lifespan in *Saccharomyces cerevisiae*, *Caenorhabditis elegans,* and *Drosophila melanogaster* [18,19,20,21]. Resveratrol is characterized by a poor bioavailability, which is mainly caused by an extensive in vivo conversion into its sulfate and gluconate metabolites [22]. Furthermore, the central double bond of resveratrol is one of the various molecular portions undergoing fast chemical and metabolic transformations [23]; therefore, we previously synthesized novel resveratrol analogues, where its double bond was replaced by an aniline scaffold [24]. Another example of a natural SIRT1 activator is quercetin (3,3′,4′,5,7-pentahydroxyflavone **2**, Figure 1), a flavone molecule widely common in fruits and vegetables, that has been proven to reduce the myocardial ischemia/reperfusion-induced cardiomyocyte apoptosis via SIRT1/peroxisome proliferator-activated receptor-γ coactivator-1α (PGC-1α) signaling in a dose-dependent manner [25]. Recent studies have demonstrated that the *Citrus* flavonoid naringenin ((2S)-4′,5,7-trihydroxyflavan-4-one **3**, Figure 1), which shares similar structural features with quercetin **2** and resveratrol, was able to slow down myocardial senescence by modulating SIRT1 [26]. Concerning synthetic SIRT1-activating compounds, SRT2104 (compound **4**, Figure 1), developed by Sirtris Pharmaceuticals, is one of the most representative examples of this class. SRT2104 is chemically characterized by an imidazothiazole core and it was studied in many clinical trials because of its beneficial effects mediated by SIRT1 activation [27,28,29,30,31,32]. Among other synthetic SIRT1 activators, 1,4-dihydropyridine-based derivative **5** (Figure 1) and naphthofuran analogue **6** (Figure 1) have also been proven to remarkably activate SIRT1 [33,34]. Very recently, our research group designed and developed bisarylaniline **7** (Figure 1), a new synthetic analogue of previously mentioned natural SIRT1 activators (compounds **1–3**, Figure 1). Indeed, compound **7** shares the same antipodal phenolic rings of natural polyphenols **1–3**, which are connected through a three-substituted anilino portion. Diarylamine **7** displays a notable SIRT1 activation ability and has been demonstrated to exert a cardioprotective effect in an ex vivo ischemia/reperfusion (I/R) model [24].

**Figure 2:**
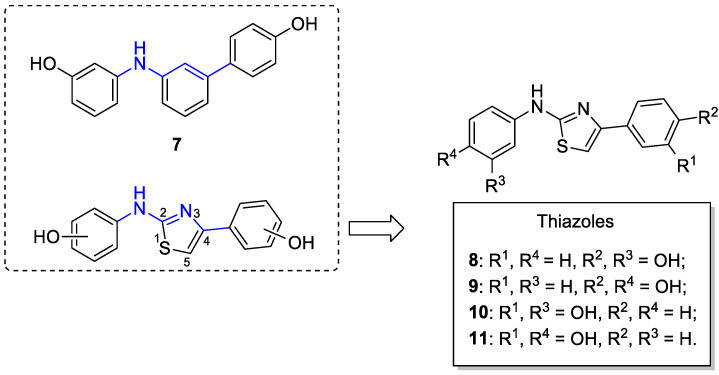
Design of small molecules activating SIRT1. The structure of previously published SIRT1 activator **7** and the general structure of the thiazole class are reported in the dashed box [24]; on the right, the specific structures of the thiazole derivatives **8–11** are reported.

Taking into account the promising results achieved in our previous work [24], we decided to further investigate the effects due to the variations in the central core of the diarylamine derivative **7**, in order to find more potent SIRT1 activators. Therefore, we decided to maintain the two pharmacophoric peripheral phenolic rings, with hydroxyl groups in the *meta* and/or *para* position, and to replace the central 1,3-disubstituted phenyl ring with a 2,4-disubstituted thiazole ring (Figure 2). The synthesized thiazoles **8–11** (Figure 2) bear a central thiazole ring that is directly connected in position 4 to one phenolic ring, whereas in position 2, it is linked to the other phenolic ring through an NH group. This modification allows for substantially conserving the same length of the linker portion between the two antipodal phenolic rings (parts of the structures highlighted in blue in the dashed panel, Figure 2); moreover, the bioisosteric replacement of the central benzene ring with the thiazole ring leads to a slight change in the spatial disposition of the two peripheral OH groups, because of the different bond angles of the central aminothiazole core. In this way, we wanted to verify if these structural changes could hopefully lead to the development of more potent SIRT1 activators. It is noteworthy to mention that compounds **8** and **9** were previously reported as allosteric inhibitors of fructose 1,6-bisphosphatase [35], and compound **11** was synthesized as an inhibitor of valosin-containing protein [36].

## 2. Results and Discussion

### 2.1. Chemistry

**Scheme 1:**
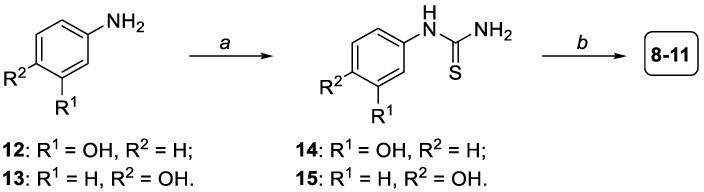
Synthesis of thiazole derivatives **8–11**. Reagents and conditions: (*a*) KSCN, conc. HCl, abs. EtOH, 110 °C, 18 h [27–41%]; (*b*) 2-bromo-4′-hydroxyacetophenone (for compounds **8** and **9**) or 2-bromo-3′-hydroxyacetophenone (for compounds **10** and **11**), abs. EtOH, MW (71 °C, 300 W, 2 min) [67–82%].

The synthesis of thiazoles **8–11** [35,36] started with the condensation between commercially available 3-aminophenol **12** or 4-aminophenol **13** and potassium thiocyanate (KSCN), in the presence of absolute ethanol as the solvent and concentrated hydrochloric acid at 110 °C overnight, so as to afford thiourea intermediates **14** and **15**, respectively (Scheme 1, step *a*). Then, intermediates **14** and **15** were reacted with 2-bromo-4′-hydroxyacetophenone to give the final thiazoles **8** and **9**, or with 2-bromo-3′-hydroxyacetophenone, thus obtaining thiazoles **10** and **11**, respectively (Scheme 1, step *b*). The Hantzsch condensation between thiourea derivatives **14** and **15** and the two commercially available bromo-hydroxyacetophenones was conducted following a microwave-assisted procedure, by using absolute EtOH as the solvent and heating at 71 °C with a power of 300 W for 2 min. Before proceeding with the biological and pharmacological evaluation of the synthesized thiazoles **8–11**, their structures were confirmed by NMR analysis (^1^H- and ^13^C-NMR), and their purity was determined by HPLC analysis and was always found to be higher than 95%.

### 2.2. Enzymatic Assays

The four final compounds **8–11** were subjected to an enzymatic assay to evaluate their ability to activate the SIRT1 enzyme. Resveratrol 100 µM (**1**, Figure 1) was used as the positive control. The activation of SIRT1 by resveratrol was set at 100% and the percentage of SIRT1 activation obtained by compounds **8–11** at 100 µM was evaluated in comparison with that of resveratrol (Table 1). In order to speculate on their potency of activation, we also reported the activation of SIRT1 shown by compound **7** (according to a previous publication [24]).

All of the tested compounds demonstrated a good ability to activate SIRT1 at the tested concentration (100 µM), superior to that of compound **7**. It is worth noting that the only derivative possessing two *meta*-OH peripheral groups (compound **10**) showed lower levels of SIRT1 activation than that of resveratrol (with about 73%, Table 1), whereas the other three analogues (**8**, **9**, and **11**), possessing at least one hydroxy group in a *para* position, all displayed activities that were about equal to resveratrol (values of about 99, 116, and 95%, respectively, Table 1). Of note, activation of the SIRT1 enzyme by compounds **8**, **9**, and **11** was generally superimposable with that of reference compound **1**. This seems to demonstrate that a minimal distance between the two terminal OH groups should be maintained for the optimal activation of SIRT1.

**Figure 3:**
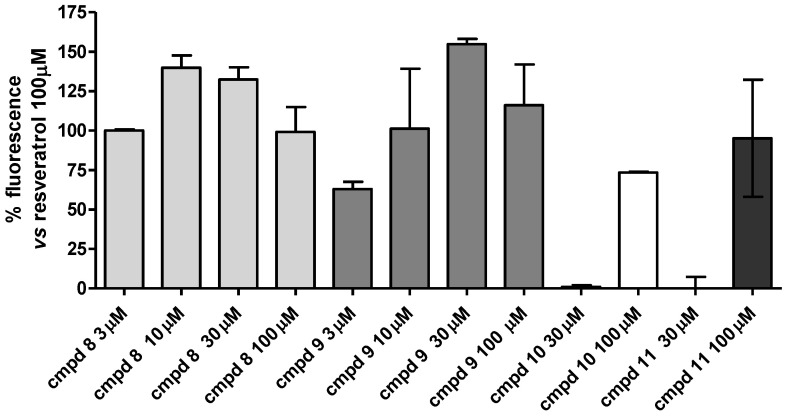
Concentration–response curve of the SIRT1 enzyme activation mediated by compounds **8**–**11**. The SIRT1 activity is expressed as % vs. the effect observed with 100 μM of resveratrol. The vertical bars symbolize the standard errors (n = 5).

To better clarify the SIRT1-activating behavior of the compounds, all of the compounds were tested at 30 and 100 µM (Figure 3), and the most potent thiazoles **8** and **9** were also evaluated at lower concentrations (3 and 10 µM, Figure 3). Compounds **8** and **9** showed a maximum activation at 10 µM and 30 µM, with an activation rate of 140 ± 8% and 155 ± 3%, respectively, thus demonstrating a higher potency than resveratrol. Conversely, for compounds **10** and **11**, at 30 µM, we observed a complete loss of activity (Figure 3). Compounds **8** and **9** displayed substantially overlapped potencies for activating the SIRT1 enzyme. For a better comparison with the previously studied compound **7**, in order to specifically verify the effect due to only the central core replacement, in vivo pharmacological investigations were carried out with compound **8**, which has the same peripheral disposition of the phenol OH groups as that in **7** (see following section).

### 2.3. In Vitro H9c2 Cytotoxicity of Compound **8**

**Figure 4:**
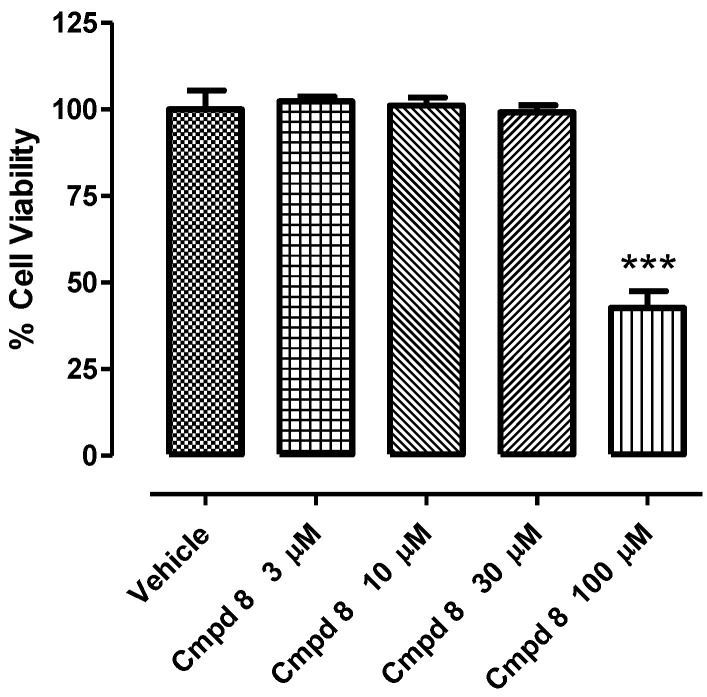
Concentration–response of compound **8** in H9c2 cell line after 24 h of treatment. Cell viability is expressed as percentage of the vehicle. One-way ANOVA followed by Bonferroni post-test was used for the statistical analysis. * represents the statistical significance vs. vehicle (*** *p* < 0.001). Bars indicate mean ± SEM.

Compound **8** was tested at 3, 10, 30, and 100 µM using rat cardio myoblast cells (H9c2) for assessing the in vitro cytotoxicity. As reported in Figure 4, Compound **8** caused a decrease in cell viability of about 50%, only when incubated at the maximum concentration (100 µM), while lower concentrations did not induce any toxic event after 24 h of treatment. This result reflects the activity of Compound **8** in the SIRT1 isolated enzyme. As a matter of fact, the maximum activation measured in the SIRT1 isolated enzyme was indeed observed when the compound was incubated at 10 µM; at this concentration, compound **8** did not cause any toxic event in vitro.

### 2.4. Cardioprotective Activity of Compound **8** in an In Vivo Acute Myocardial Infarct Model

**Figure 5:**
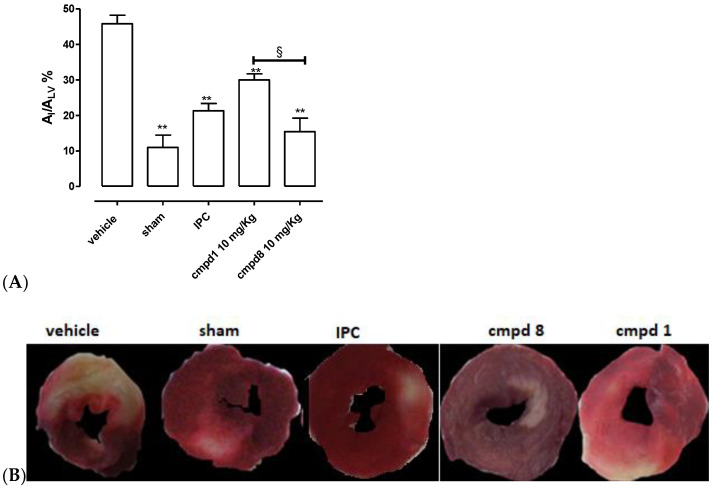
(**A**) The histograms show the extent of the ischemic area (A_I_) compared with the total area of the left ventricle (A_LV_) in the different groups. Data are expressed as mean ± SEM. *indicates significant difference vs. vehicle (** *p* < 0.01). § indicates significant difference vs. compound **1** (resveratrol) group (§ *p* < 0.05). Number of animals used = 5. (**B**) Exemplificative pictures of several types of treatment groups.

SIRT1 is a well-described target to effectively obtain anti-ischemic cardioprotective effects [19,24,37,38,39]. The procedure of in vivo coronary artery occlusion/reperfusion led to reproducible damage at the myocardium level; indeed, the ischemic area was 46 ± 2 % of the whole area of the left ventricle (A_I_/A_LV_, Figure 5). In the sham animals, which were submitted to the experimental protocol of acute myocardial infarct, but without occlusion/reperfusion, the extension of A_I_/A_LV_ was negligible (11 ± 2%, Figure 5). Moreover, the ischemic preconditioning (IPC) protocol induced a significant reduction in ischemic injury (A_I_/A_LV_ = 21 ± 2%, Figure 5). The pharmacological pretreatment with the reference compound **1** (10 mg/Kg) led, according to literature [24], to a significant reduction in the injured areas (A_I_/A_LV_ = 30 ± 2%; Figure 5), compared with the vehicle. Finally, animals pre-administered with compound **8** (at the same dose of resveratrol) showed more evident cardioprotection compared with the vehicle; of note, compound **8** was able to promote a higher level of protection than that of resveratrol; indeed, the extension of the ischemic area was 15 ± 4% (Figure 5), confirming the validity of our molecular design for introducing a thiazole ring in the central core of **7**.

### 2.5. Molecular Modeling Studies

**Figure 6:**
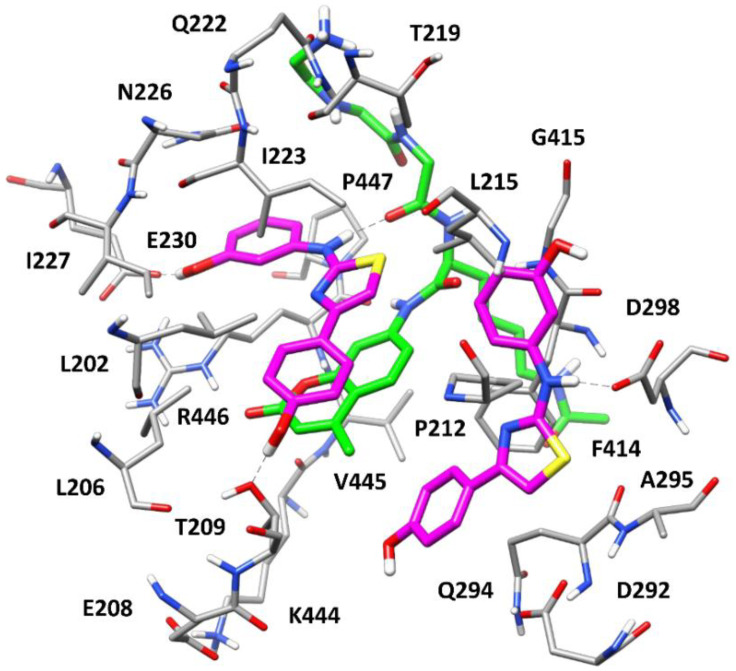
Minimized average structure of SIRT1 in complex with p53-AMC peptide (green) and two molecules of compound **8** (purple). The ligand–protein and ligand–peptide H-bonds are highlighted as black dashed lines. For clarity, the side chains of R1, H2, and K3 of p53-AMC are not shown.

With the aim of evaluating the potential binding mode of the new series of compounds within SIRT1, docking and molecular dynamic (MD) simulation studies were performed using representative compound **8** and the X-ray structure of SIRT1 in complex with p53-AMC peptide and resveratrol (PDB code 5BTR [40]) as a reference. The same docking approach and MD protocol, validated and applied in our previous work [24], were employed for this study. In particular, the ligand was initially docked into the three subpockets (referred to as site 1, 2, and 3) identified within the p53-AMC-bound SIRT1 catalytic site and occupied by the three molecules of resveratrol in the reference complex. As found for parent diarylamine **7**, only two molecules of compound **8** were predicted to interact with SIRT1, as two of the three docking results converged into a single binding conformation occupying site 2 and part of site 3. The corresponding SIRT1/p53-AMC/**8** quaternary complex predicted by the docking was then analyzed through the validated 500 ns MD protocol. Figure 6 shows the structure of the complex refined by the MD simulation. The ligand molecule occupying site 1 is located above and parallel to the coumarin core of the p53-AMC peptide, which mainly constitutes the “floor” of site 1 and establishes extensive π–π stacking interactions with the arylthiazole moiety of the ligand. The compound is predominantly anchored to this portion of the SIRT1 catalytic site thanks to an H-bond with the backbone carbonyl group of p53-AMC directed inside the pocket and a charge-assisted H-bond with E230, which are formed by the *m*-OH-anilino moiety of the ligand and are maintained for most of the MD simulation. Additionally, a transient H-bond between T209 and the *p*-OH-phenyl ring of the ligand is also observed. The arylthiazole moiety of **8** shows multiple hydrophobic interactions with residues delimiting site 1, mainly L206, P212, L215, and I223, as well as with the other ligand molecules located in site 2. Moreover, the *m*-OH-phenyl ring of the ligand predominantly forms π-based interactions with the amide group of N226 and lipophilic interactions with the side chains of R446 and P447. The second molecule of the ligand almost fully occupies site 2 with its arylthiazole moiety, forming a π–π stacking with F414 and hydrophobic interactions mainly with Q294, A295, and the coumarin core of the p53-AMC peptide. The NH-group of the ligand forms a stable charge-assisted H-bond with D298, which anchors the compound to the pocket, while none of the two OH groups seems to establish relevant interactions with the binding site residues. Moreover, the *m*-OH-phenyl ring of the ligand occupies part of site 3, forming hydrophobic interactions with P212, L215, and G415 and with the thiazole ring of the other ligand molecule located in site 1.

As previously predicted for the parent compound **7**, our modeling studies suggest that the synthesized ligand **8** is able to mimic most of the interactions with SIRT1 and with the bound p53-AMC peptide that were observed for resveratrol, as well as to stably occupy most of the catalytic pocket of the enzyme in a conformation that induced an agonist activity. Notably, although the two molecules of compound **8** show binding conformations quite similar to those predicted for parent compound **7**, remarkable differences could be identified. In fact, the OH group present on the arylthiazole moiety of **8** forms only a transient H-bond with T209, which is consistent with the moderate reduction in agonist activity observed in compound **10**, which presents a *m*-OH group on the same ring and thus cannot form such an interaction. On the contrary, the OH group of the *m*-OH-anilino moiety of compound **8** strongly interacts with E230, as observed for resveratrol but not for compound **7**, while it does not interact with D298, as predicted for **7**. This feature is in agreement with the SAR data, showing that moving the OH group of **8** from the *meta* to the *para* position is not detrimental to the agonist activity of the ligands; on the contrary, this can actually increase the activity (as in compound **9**) or balance the potency drop due to the position shift of the other OH group in the arylthiazole moiety (as in **11**). In fact, by superimposing the herein predicted SIRT1/p53-AMC/**8** quaternary complex with the SIRT1/p53-AMC/resveratrol complex refined through MD simulations in our previous work (Figure S9) [24], it is possible to verify that the same interaction with E230 may be formed by a *p*-OH-anilino moiety, as in compound **9**, which would actually even better mimic the H-bond observed between resveratrol and E230. Overall, these considerations strongly support the reliability of the binding mode predicted for compound **8** and its congeners, and allows for a rational structure-based interpretation of the preliminary SAR data experimentally derived for this new compound series.

## 3. Materials and Methods

### 3.1. Synthesis. General Procedures and Materials

All of the solvents and chemicals were used as purchased, without further purification. Chromatographic separations were performed on silica gel columns by flash chromatography (silica gel pore size 60 Å, 40–63 μm particle size). Reactions were followed by thin layer chromatography (TLC) on Merck aluminum silica gel (60 F254) sheets that were visualized under a UV lamp. Evaporation was performed in vacuo (rotating evaporator). Sodium sulfate was always used as the drying agent. The proton (^1^H) and carbon (^13^C) NMR spectra were obtained with a Bruker Avance III 400 MHz spectrometer using the indicated deuterated solvents. Chemical shifts were given in parts per million (ppm) (δ relative to residual solvent peak for ^1^H and ^13^C). The ^1^H-NMR spectra were reported in this order: multiplicity and number of protons. Standard abbreviation indicating the multiplicity were used as follows: s = singlet, dd = doublet of doublets, ddd = doublet of doublet of doublets, t = triplet, dt = doublet of triplets, m = multiplet, and bs = broad singlet. Microwave-assisted reactions were run in a Biotage® Initiator^+^ microwave synthesizer. HPLC analysis was used to determine the purity: all of the target compounds (i.e., assessed in biological assays) were ≥ 95% pure by HPLC, as confirmed via UV detection (λ = 254 nm). Analytical reversed-phase HPLC was conducted using a Kinetex EVO C18 column (5 μm, 150 × 4.6 mm, Phenomenex, Inc.); eluent A, water; eluent B, MeOH; after 5 min at 25% B, a gradient was formed from 25% to 75% of B over 5 min, and was held at 75% of B for 10 min; the flow rate was 1 mL/min. The HPLC analyses were performed at 254 nm. The ESI-MS spectra were recorded by direct injection at a 5 μl min^−1^ flow rate in an Orbitrap Q Exactive Plus high-resolution mass spectrometer (Thermo Fischer Scientific Inc., Germany), equipped with an HESI source. The working conditions were as follows: negative polarity, spray voltage 3200 V, capillary temperature 290 °C, S-lens RF level 50, the sheath gas was set at 28, and the auxiliary gas was set at 4 (arbitrary units). For acquisition and analysis, Xcalibur 4.2 software (Thermo) was used. For spectra acquisition, a nominal resolution (at *m/z* 200) of 140,000 was used. Yields refer to the isolated and purified products derived from non-optimized procedures.

#### 3.1.1. General Procedure for the Formation of Compounds **14**,**15**

HCl 37% (0.58 mL) was added dropwise to a stirred solution of 3-aminophenol **12** or 4-aminophenol **13** (4.58 mmol, 500 mg) in absolute ethanol (4 mL). The suspension that formed was heated to reflux and when all of the suspension had dissolved, then potassium thiocyanate (6.87 mmol) was added. The reaction was stirred at 110 °C for 18 h. After cooling the reaction mixture at room temperature, the solution was diluted with water, repeatedly extracted with EtOAc, dried over Na_2_SO_4_, and the solvent was evaporated. The residue was purified by column chromatography, and was eluted by *n*-hexane/EtOAc mixtures as the eluent, to give thiourea derivatives **14** and **15**, according to the methods reported in the literature [35,41].

1-(3-Hydroxyphenyl)thiourea (**14**): 41% yield from 3-aminophenol **12**. ^1^H-NMR (DMSO-*d_6_*) δ (ppm): 6.52 (ddd, 1H, *J* = 8.1, 2.3, 0.8 Hz), 6.72–6.79 (m, 1H), 6.85–6.91 (m, 1H), 7.09 (t, 1H, *J* = 8.0 Hz), 7.43 (bs, 2H), 9.45 (s, 1H), and 9.57 (s, 1H).

1-(4-Hydroxyphenyl)thiourea (**15**): 27% yield from 3-aminophenol **13**. ^1^H-NMR (DMSO-*d_6_*) δ (ppm): 6.71 (AA’XX’, 2H, *J_AX_* = 8.7 Hz, *J_AA’/XX’_* = 2.7 Hz), 7.02-7.09 (m, 2H), 7.20 (bs, 2H), 9.35 (s, 1H), and 9.37 (s, 1H).

#### 3.1.2. General Procedure for the Formation of Compounds **8**–**11**

A solution of compound **14** or **15** (0.594 mmol, 100 mg) and 2-bromo-4′-hydroxyacetophenone (for compounds **8** and **9**) or 2-bromo-3′-hydroxyacetophenone (for compounds **10** and **11**) (0.594 mmol) in 3.5 mL of ethanol in a sealed vial was placed in a microwave reactor and was subjected to MW irradiation at 71 °C for 2 min (300 W). The reaction mixture was cooled to room temperature, the the residue was diluted with water and was extracted with EtOAc. The organic phase was dried over Na_2_SO_4_, and the solvent was removed under reduced pressure. The residue was purified with flash column chromatography (silica gel, mixtures of *n*-hexane/EtOAc) and pure fractions containing the desired compound were evaporated to dryness, affording final compounds **8**–**11**. Experimental data are in agreement with those reported in literature for compounds **8** and **9** [35], no data are reported for compound **11** [36]. The same procedure [35,36] was used for the synthesis of the new compound **10**.

3-((4-(4-Hydroxyphenyl)thiazol-2-yl)amino)phenol (**8**): 82% yield from 2-bromo-4′-hydroxyacetophenone and **14**, yellow solid. ^1^H-NMR (DMSO-*d_6_*) δ (ppm): 6.36 (ddd, 1H, *J* = 8.0, 2.3, 1.0 Hz), 6.81 (AA’XX’, 2H, *J_AX_* = 9.1 Hz, *J_AA’/XX’_* = 2.4 Hz), 7.00 (ddd, 1H, *J* = 8.0, 2.0, 1.0 Hz), 7.03 (s, 1H), 7.09 (t, 1H, *J* = 8.0 Hz), 7.32 (t, 1H, *J* = 2.1 Hz), 7.75 (AA’XX’, 2H, *J_AX_* = 8.7 Hz, *J_AA’/XX’_* = 2.4 Hz), 9.39 (s, 1H), 9.53 (s, 1H), and 10.06 (s, 1H). ^13^C-NMR (DMSO-*d_6_*) δ (ppm): 99.65, 103.97, 107.79, 108.35, 115.28 (2C), 126.00, 127.12 (2C), 129.56, 142.31, 150.38, 157.06, 157.91, and 162.80. HPLC analysis: retention time = 12.635; peak area 98% (254 nm). HRMS: *m/z* for C_15_H_11_N_2_O_2_S [M-H]^−^ calculated: 283.05412, found: 283.05478.

4-(2-((4-Hydroxyphenyl)amino)thiazol-4-yl)phenol (**9**): 77% yield from 2-bromo-4′-hydroxyacetophenone and **15**, yellow solid. ^1^H-NMR (DMSO-*d_6_*) δ (ppm): 6.74 (AA’XX’, 2H, *J_AX_* = 8.9 Hz, *J_AA’/XX’_* = 2.8 Hz), 6.79 (AA’XX’, 2H, *J_AX_* = 8.7 Hz, *J_AA’/XX’_* = 2.3 Hz), 6.93 (s, 1H), 7.46 (AA’XX’, 2H, *J_AX_* = 8.9 Hz, *J_AA’/XX’_* = 2.7 Hz), 7.69 (AA’XX’, 2H, *J_AX_* = 8.6 Hz, *J_AA’/XX’_* = 2.4 Hz), 9.08 (s, 1H), 9.51 (s, 1H), and 9.82 (s, 1H). ^13^C-NMR (DMSO-*d_6_*) δ (ppm): 98.72, 115.28 (2C), 115.45 (2C), 119.13 (2C), 126.14, 127.02 (2C), 133.43, 150.34, 152.21, 156.98, and 164.02. HPLC analysis: retention time = 12.419; peak area 97% (254 nm). HRMS: *m/z* for C_15_H_11_N_2_O_2_S [M-H]^−^ calculated: 283.05412, found: 283.05481.

3-(2-((3-Hydroxyphenyl)amino)thiazol-4-yl)phenol (**10**): 79% yield from 2-bromo-3′-hydroxyacetophenone and **14**, white solid. ^1^H-NMR (DMSO-*d_6_*) δ (ppm): 6.35–6.41 (m, 1H), 6.71 (ddd, 1H, *J* = 8.0, 2.5, 0.9 Hz), 7.06–7.12 (m, 2H), 7.17-7.23 (m, 3H), 7.32 (t, 1H, *J* = 1.9 Hz), 7.33–7.37 (m, 1H), 9.40 (s, 1H), 9,46 (s, 1H), 10.09 (s, 1H). ^13^C-NMR (DMSO-*d_6_*) δ (ppm): 102.64, 104.02, 107.84, 108.49, 112.66, 114.61, 116.64, 129.50, 129.58, 135.86, 142.25, 150.26, 157.50, 157.90, and 162.85. HPLC analysis: retention time = 12.827; peak area 96% (254 nm). HRMS: *m/z* for C_15_H_11_N_2_O_2_S [M-H]^−^ calculated: 283.05412, found: 283.05475.

3-(2-((4-Hydroxyphenyl)amino)thiazol-4-yl)phenol (**11**): 67% yield from 2-bromo-3′-hydroxyacetophenone and **15**, white solid. ^1^H-NMR (DMSO-*d_6_*) δ (ppm): 6.69 (ddd, 1H, *J* = 8.0, 2.1, 1.3 Hz), 6.75 (AA’XX’, 2H, *J_AX_* = 8.9 Hz, *J_AA’/XX’_* = 2.8 Hz), 7.11 (s, 1H), 7.19 (t, 1H, *J* = 7.8 Hz), 7.27–7.34 (m, 2H), 7.47 (AA’XX’, 2H, *J_AX_* = 8.9 Hz, *J_AA’/XX’_* = 2.8 Hz), 9.12 (s, 1H), 9.44 (s, 1H), and 9.87 (s, 1H). ^13^C-NMR (DMSO-*d_6_*) δ (ppm): 101.71, 112.69, 114.50, 115.44 (2C), 116.46, 119.18 (2C), 129.50, 133.36, 135.98, 150.19, 152.28, 157.51, and 163.99. HPLC analysis: retention time = 12.620; peak area 97% (254 nm). HRMS: *m/z* for C_15_H_11_N_2_O_2_S [M-H]^−^ calculated: 283.05412, found: 283.05478.

### 3.2. In Vitro Screening on Isolated SIRT1 Enzyme

The effects of the new compounds on the SIRT1 activity were evaluated by a direct enzymatic assay using a SIRT1 Direct Fluorescent Screening Assay Kit (Cayman Chemical, Ann Arbor, MI, USA), following the protocol user guide. Before performing the test, the possible interferences of the compounds under examination with the fluorophore and/or developer were evaluated according to the manufacturer’s protocol. Briefly, the fluorescence was analyzed with an EnSpire spectrofluorometer (PerkinElmer, Waltham, MA, USA) at a wavelength of 350–360 nm in excitation and 450–465 nm in emission. The increase in recorded fluorescence was directly proportional to the activation of SIRT1. The obtained data were analyzed by removing the baseline and normalizing to the fluorescence value vs. vehicle (DMSO 0.1%; corresponding to 0%) and the SIRT1 activator of 100 μM of resveratrol (named compound **1** (Sigma-Aldrich, St. Louis, MO, USA), which was assigned the value of 100%.

### 3.3. In Vitro H9c2 Toxicity of Compound **8**

Rat cardiomyocyte (H9c2) (ATCC, Manassas, VA, USA) cells were cultured in DMEM supplemented with 10% fetal bovine serum, 100 U/ml of penicillin, and 100 mg/ml of streptomycin at 37 °C in a 5% CO_2_ humidified atmosphere. The cell culture media were changed every 2–3 days, and the cells were sub-cultured once they reached 70–80% confluence.

Cell viability was assessed using the WST1 assay. Briefly, 10^4^ cells were seeded in 96-well plates and were treated with four different concentrations of compound **8** (3, 10, 30, and 100 µM) or vehicle (DMSO 0.1%) for 24 h. At the end of each treatment, cell viability was assessed using the cell proliferation reagent WST-1 (4-[3-(4-iodophenyl)-2-(4-nitrophenyl)-2H-5-tetrazolium]-1,3-benzene disulphonate) (Roche, Basel, Switzerland), which was cleaved to formazan in living cells. WST-1 was added at 1:10 of the total volume of wells and after 60 min of incubation at 37 °C, the absorbance was measured at 450 nm with a multiplate reader (Enspire, Perkin-Elmer, Waltham, MA, USA). Experiments were performed in triplicate and were repeated at least three times. Data analysis for concentration–response experiments was performed using GraphPad Prism 5 (GraphPad Software, La Jolla, CA, USA), and the data were normalized with respect to the vehicle values, representing 100% cell growth.

### 3.4. In Vivo Acute Myocardial Infarction

All of the procedures were performed according to European (EEC Directive 2010/63) and Italian (D.L. 4 March 2014 n. 26) legislation (protocol number 909/2016/PR, 29 June 2016). Adult male Wistar rats, 350–400 g, were housed in humidity and temperature-controlled rooms (22 °C and 50%, respectively) with food and water ad libitum, and exposed to 12 h:12 h light/dark cycles. The experimental protocol for coronary occlusion/reperfusion was performed as previously described [42,43], with minor modifications. Two hours before the experimental procedures, rats received an i.p. injection of compounds **1** and **8** (both at 10 mg/kg) or the vehicle (DMSO, 1 ml/kg). Then, the rats were anaesthetized with sodium pentobarbital (70 mg/kg, i.p.). The trachea was intubated and connected to a rodent ventilator (mod. 7025 Ugo Basile, Comerio, Italy) for artificial ventilation with room air (stroke volume, 1 ml/100 g body weight; 70 strokes/min). The electrocardiogram (ECG) was continuously measured by lead II (Mindray, PM5000, 2 Biological Instruments, Varese-Italy). The chest was opened by a left thoracotomy. A 6-0 surgical needle was passed around the left anterior descending coronary artery (LAD), located between the base of the pulmonary artery and left atrium. The ends of the suture were passed through a polypropylene tube (PE50) to form a snare. To induce infarction, LAD was occluded by pulling the snare and then fixing it in place by clamping the tube with a hemostat. When the snare was released, the reperfusion was initiated. The acute infarct protocol consisted of 30 min of occlusion/120 min of reperfusion; successful occlusion was confirmed by observing regional cyanosis downstream of the ligature, and by ST elevation in the ECG recording. A group of vehicle-pretreated animals was submitted to an IPC procedure, achieved by three cycles of 5 min of occlusion/10 min of reperfusion, followed by 30 min of coronary occlusion and 120 min of reperfusion. Some of the animals were assigned to the sham group, i.e., submitted to all surgical procedures without occlusion/reperfusion. Each experimental group was composed of five animals. At the end of reperfusion, the rats were euthanized by an overdose of pentobarbital sodium, and then the hearts were quickly excised, mounted on a Langendorff apparatus (Radnoti, Covina, CA, USA), and perfused for about 10 min with Krebs solution at 37 °C to wash out the coronary blood vessels. Then, the atria and right ventricle were removed from the hearts, and the left ventricular tissue was dried, frozen at −20 °C, and cut into 4–5 transverse slices from the apex to base at an equal thickness (about 2 mm). The slices were then incubated in a 1% 2,3,5-triphenyltetrazolium chloride (TTC) solution in a phosphate buffer (pH 7.4) at 37 °C for 20 min. TTC reacted with NADH in the presence of dehydrogenase enzymes, to form a formazan derivative, which stains the viable cells with an intense red color. Then, the slices were fixed overnight in 10% formaldehyde and, finally, they were photographed. The infarct area (A_I_/A_LV_) was planimetrically evaluated using an image analyzer program (The GIMP 2). Student’s t test was selected for the statistical analysis, and the difference between groups was considered statistically different when *p* < 0.05.

### 3.5. Docking Calculations

The compounds were built with Maestro and then subjected to energy minimization performed with Macromodel, until a convergence value of 0.05 kcal/Å•mol was achieved, by employing the CG algorithm, MMFFs force field, and a distance-dependent dielectric constant of 1.0. The compounds were docked into the X-ray structure of SIRT1 in complex with the p53-AMC peptide and resveratrol (PDB code 5BTR [40]). All of the docking calculations were performed with GOLD software, using the ChemScore fitness function. For each docked compound, we performed three different docking studies related to the three different receptor sub-sites occupied by the three resveratrol molecules in the reference X-ray complex. In each calculation, the docking site included all of the residues that strayed within a 10 Å shell from the specific bound ligand in the reference X-ray complex. The compounds were subjected to 100 genetic algorithm runs, in which the “allow early termination” option was deactivated, while the possibility for the ligand to flip ring corners was activated. All of the other settings were left as their defaults. The root mean-squared deviation (RMSD) threshold for pose clustering was set to 2.0 Å. The best docked conformation belonging to the best cluster of solutions (top-scored pose) was considered for each ligand in each docking study. By following this procedure, resveratrol was properly self-docked in each of the three receptor sub-sites, with RMSD values between each top-scored pose and the corresponding experimental disposition below 2.0 Å, thus validating the reliability of the protocol. Two out of the three the top-scored poses obtained for each of the phenolic derivatives of **10** and **11** converged in a single binding mode. For this reason, the final corresponding SIRT1–peptide–ligand complexes predicted by docking included only two ligand molecules.

### 3.6. Molecular Dynamics Simulations

MD simulations were carried out using AMBER, version 20, using the ff14SB force field. General AMBER force field (GAFF) parameters were used for the ligand, whose partial charges were assigned using the Antechamber suite of AMBER 20, based on the AM1-BCC method. The reference SIRT1–peptide–resveratrol complex (PDB code 5BTR) and the two predicted SIRT1–peptide–ligands complexes were placed at the centre of a rectangular parallelepiped box and solvated with a 15 Å water cap, generated using TIP3P explicit solvent model. Sodium ions were added to neutralize the solvated systems, which were then energy minimized using a two-step protocol. In the first step, only the minimization of the solvent was performed by applying a harmonic restraint of 100 kcal/mol•Å^2^ on all of the solute atoms. In the second step, 5000 cycles of steepest descent followed by conjugate gradient (CG) were used to minimize the whole system, until a reaching aconvergence of 0.05 kcal/Å•mol. The minimized complexes were used as input structures for the MD simulations, which were run using Particle Mesh Ewald (PME) electrostatics, with a cut-off of 10 Å for the non-bonded interactions and periodic boundary conditions. The SHAKE algorithm was used to constrain all of the bonds involving hydrogen atoms and a time step of 2.0 fs was thus used for the simulations, following a protocol optimized from previous studies [44]. Initially, a heating stage of 1 ns, in which the temperature of the system was raised from 0 to 300 K, was performed using constant-volume periodic boundary conditions. An initial equilibration stage of constant-pressure periodic boundary MD was run for 3 ns, keeping the temperature of the system at a constant value of 300 K using the Langevin thermostat. A second, longer constant-pressure MD (20 ns) was then performed at 300 K for assuring the equilibration of the protein–peptide–ligand binding conformation. Finally, a 500 ns production step was performed, maintaining the same constant pressure and temperature conditions. All α carbons of the protein were subjected to a harmonic potential of 10 kcal/mol•Å^2^ during all MD stages, for a total of 524 ns of simulation. The final structures of the different complexes corresponded to the average of the last 250 ns of MD simulation minimized by the CG method until a convergence of 0.05 kcal/mol•Å^2^. The average structures were obtained using the Cpptraj program implemented in AMBER 20.

## 4. Conclusions

The SIRT1 enzyme has been widely described as a challenging target for multiple strategies addressed for the prevention/treatment of several chronic age-related diseases, including myocardial infarct [16,19,37,38,39,45]. Resveratrol is considered to be one of the reference SIRT1 activators. However, its unfavorable pharmacokinetic profile has prompted research into novel agents able to positively modulate SIRT1 activity and displaying better overall profiles. In this context, in order to improve the SIRT1 activation of a previously identified chemical class of diarylamine derivatives [24], the central benzene ring of the original scaffold of this series was replaced by a bioisosteric thiazole ring. This structural modification turned out to be successful, as the compounds [35,36] showed their SIRT1 activating abilities to be similar or superior to those of resveratrol, and had better in vivo activities when compared with those of the original diarylamine derivatives. In particular, the selected compound **8** were proven to reduce the myocardial damage induced by an in vivo occlusion/reperfusion event at a relatively low dose, thus demonstrating the effective cardioprotective profile of this derivative. Noteworthy, the cardioprotection induced by compound **8** was significantly greater than resveratrol and of diarylamine derivatives, leading us to identify more efficient thiazole-based SIRT1 activators. Furthermore, the in silico approach highlights the putative mechanism through which compound **8** and its congeners, similarly to resveratrol, can mimic the interactions with SIRT1 and with the bound p53-AMC peptide. Further studies will be implemented for an evaluation of the systemic pharmacokinetic and in vivo toxicity profiles of these derivatives. Indeed, as reported by Suzuki and colleagues, SIRT1 activation might facilitate tumour cell migration across vascular endothelial cell layer, stimulating the increase in VEGF levels, thereby promoting metastasis through enhanced angiogenesis [46].

## Figures and Tables

**Table 1:** Percentage of SIRT1 activation of thiazoles **8–11** compared with that of resveratrol (**1**) and compound **7**.

Compound	% SIRT1 Activation
Resveratrol (**1**)	100
**7**	62.7 ± 2.7
**8**	99.1 ± 15.8
**9**	116.0 ± 25.9
**10**	73.4 ± 0.5
**11**	95.1 ± 37.1

## Data Availability

Data are contained within the article and Supplementary Materials.

## Supplementary Materials

The following supporting information can be downloaded at: https://www.mdpi.com/article/10.3390/molecules27196535/s1. Figures S1–S4: RP-HPLC traces of the final compounds; Figures S5–S12: NMR spectra of the final compounds; Figure S13: Superimposition of SIRT1/p53-AMC/**8** and SIRT1/p53-AMC/resveratrol complexes; Figures S14–S17: ESI-HRMS spectra of the final compounds.
