# Supplementary material for: Sirtuin 1-Activating Compounds: Discovery of a Class of Thiazole-Based Derivatives
Source: Molecules. 2022 Oct 3;27(19):6535. doi: 10.3390/molecules27196535 (PMC9570679; doi:10.3390/molecules27196535)
Supplement: Supplementary file 1 [file molecules-27-06535-s001.zip › molecules-1862938-supplementary.pdf]

Supporting Information

Sirtuin 1-activating compounds: discovery of a class of thiazole-based derivatives

Giulia Bononi, Valentina Citi, Margherita Lapillo, Alma Martelli, Giulio Poli, Tiziano Tuccinardi, Carlotta Granchi, Lara Testai, Vincenzo Calderone, Filippo Minutolo

Table of Contents

Figure S1-S4. RP-HPLC traces of the final compounds. S2-S5

Figure S5-S12. ^{1}H and ^{13}C NMR spectra of the final compounds. S6-S13

Figure S13. Superimposed SIRT1/p53-AMC/**8** and SIRT1/p53-AMC/resveratrol complexes S14

Figure S14-S17. ESI-HRMS spectra of the final compounds. S15-S16

==== Shimadzu LabSolutions Analysis Report ====

Sample Name : ML13_500uM
Sample ID : ML13_500uM
Data Filename : ML13_500uM_new_p.lcd
Method Filename : MAGL254 MeOH.lcm

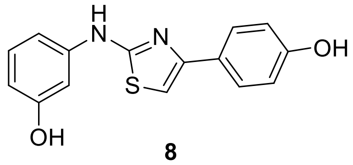

8

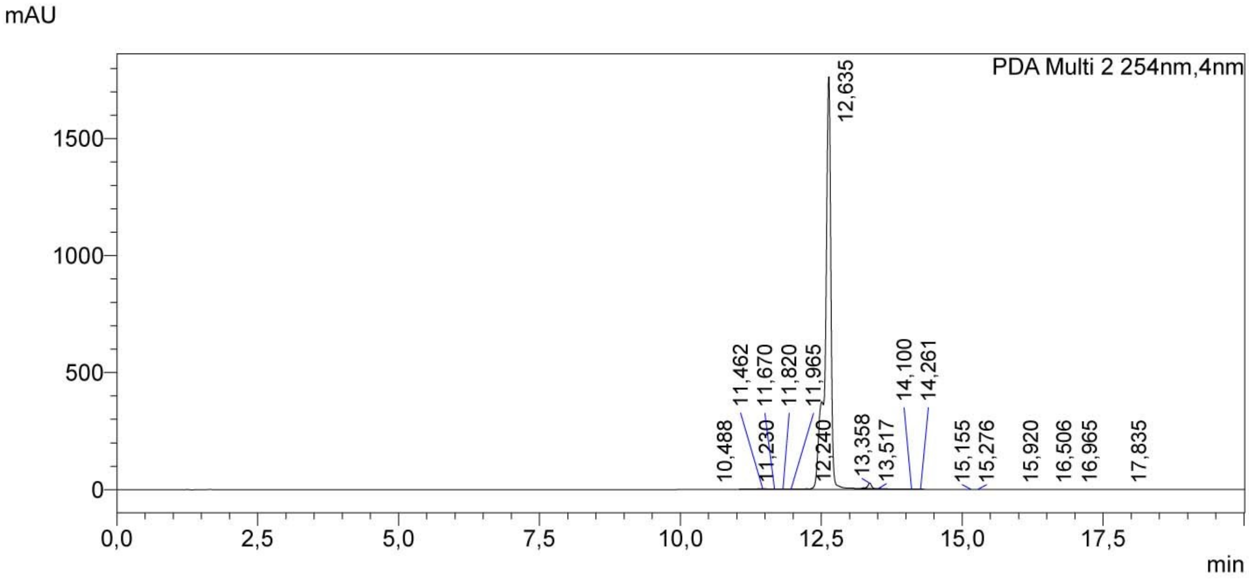

PDA Ch2 254nm

Peak#	Ret. Time	Area	Area%	Height
1	10,488	1987	0,015	196
2	11,230	27128	0,208	1682
3	11,462	37186	0,285	2853
4	11,670	10098	0,077	1332
5	11,820	10725	0,082	1446
6	11,965	8543	0,065	1106
7	12,240	21470	0,165	2233
8	12,635	12776722	97,934	1761991
9	13,358	124767	0,956	23637
10	13,517	15096	0,116	1891
11	14,100	1604	0,012	215
12	14,261	1066	0,008	199
13	15,155	2167	0,017	381
14	15,276	1791	0,014	244
15	15,920	1106	0,008	160
16	16,506	1519	0,012	165
17	16,965	1011	0,008	135
18	17,835	2283	0,017	225
Total		13046268	100,000	1800092

Figure S1. HPLC chromatogram of compound **8**.

==== Shimadzu LabSolutions Analysis Report ====

Sample Name : ML19_500uM
Sample ID : ML19_500uM
Data Filename : ML19_500uM_p.lcd
Method Filename : MAGL254 MeOH.lcm

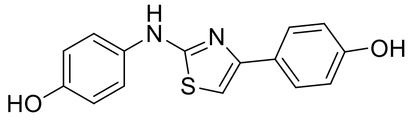

9

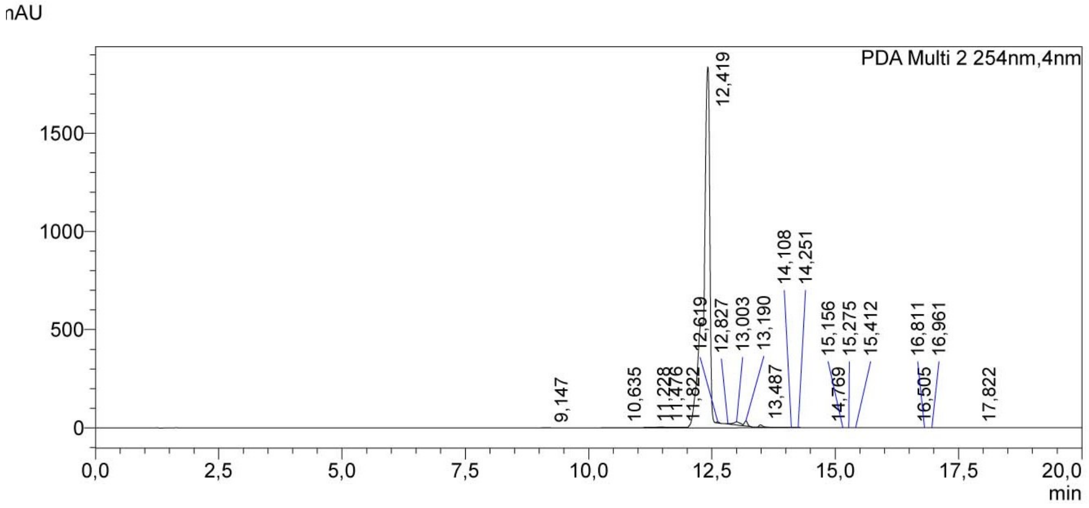

PDA Ch2 254nm

Peak#	Ret. Time	Area	Area%	Height
1	9,147	13155	0,073	1024
2	10,635	14171	0,079	588
3	11,228	46352	0,259	2183
4	11,476	68848	0,384	3541
5	11,822	14998	0,084	2036
6	12,419	17399929	97,042	1837386
7	12,619	16546	0,092	5294
8	12,827	17410	0,097	3074
9	13,003	154593	0,862	15585
10	13,190	110489	0,616	26022
11	13,487	57580	0,321	12196
12	14,108	1266	0,007	249
13	14,251	1416	0,008	229
14	14,769	1377	0,008	183
15	15,156	2365	0,013	363
16	15,275	1744	0,010	279
17	15,412	2017	0,011	266
18	16,505	1871	0,010	186
19	16,811	1092	0,006	117
20	16,961	1254	0,007	183
21	17,822	1889	0,011	207
Total		17930361	100,000	1911189

Figure S2. HPLC chromatogram of compound **9**.

==== Shimadzu LabSolutions Analysis Report ====

Sample Name : ML12_500uM
Sample ID : ML12_500uM
Data Filename : ML12_500uM_p.lcd
Method Filename : MAGL254_MeOH.lcm

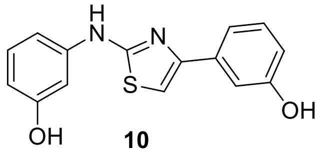

10

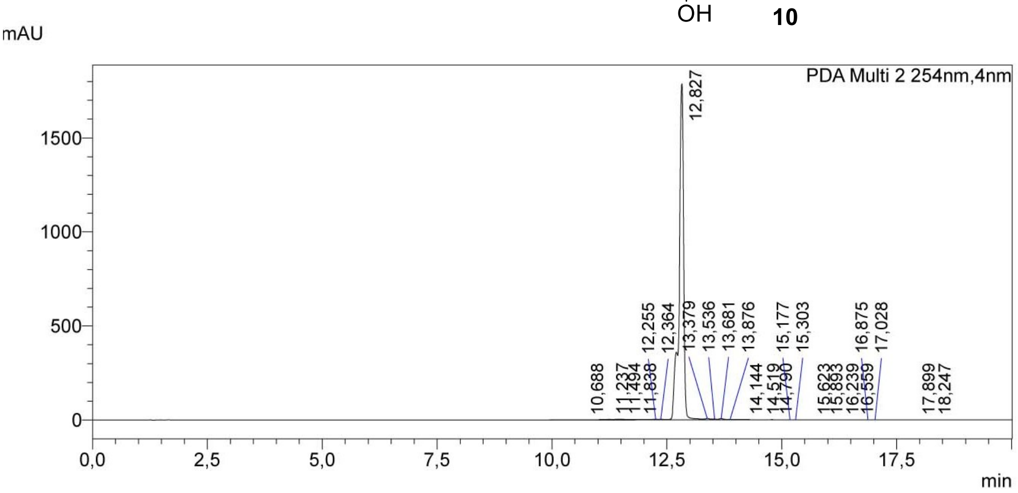

PDA Ch2 254nm

Peak#	Ret. Time	Area	Area%	Height
1	10,688	36310	0,285	986
2	11,237	54828	0,431	2860
3	11,494	71957	0,566	3999
4	11,838	15904	0,125	2177
5	12,255	55607	0,437	3912
6	12,364	23705	0,186	3350
7	12,827	12181735	95,754	1786858
8	13,379	81858	0,643	7202
9	13,536	19705	0,155	4093
10	13,681	57558	0,452	6777
11	13,876	25795	0,203	2111
12	14,144	24085	0,189	1417
13	14,519	11058	0,087	906
14	14,790	14473	0,114	1025
15	15,177	7570	0,060	836
16	15,303	7919	0,062	732
17	15,623	6683	0,053	416
18	15,893	5971	0,047	330
19	16,239	4318	0,034	398
20	16,559	5752	0,045	449

Peak#	Ret. Time	Area	Area%	Height
21	16,875	2905	0,023	298
22	17,028	2590	0,020	357
23	17,899	2423	0,019	241
24	18,247	1212	0,010	124
Total		12721924	100,000	1831854

Figure S3. HPLC chromatogram of compound **10**.

==== Shimadzu LabSolutions Analysis Report ====

Sample Name : ML20_500uM
Sample ID : ML20_500uM
Data Filename : ML20_500uM_p.lcd
Method Filename : MAGL254 MeOH.lcm

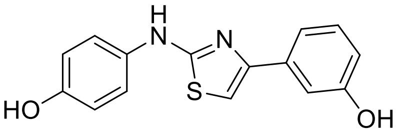

11

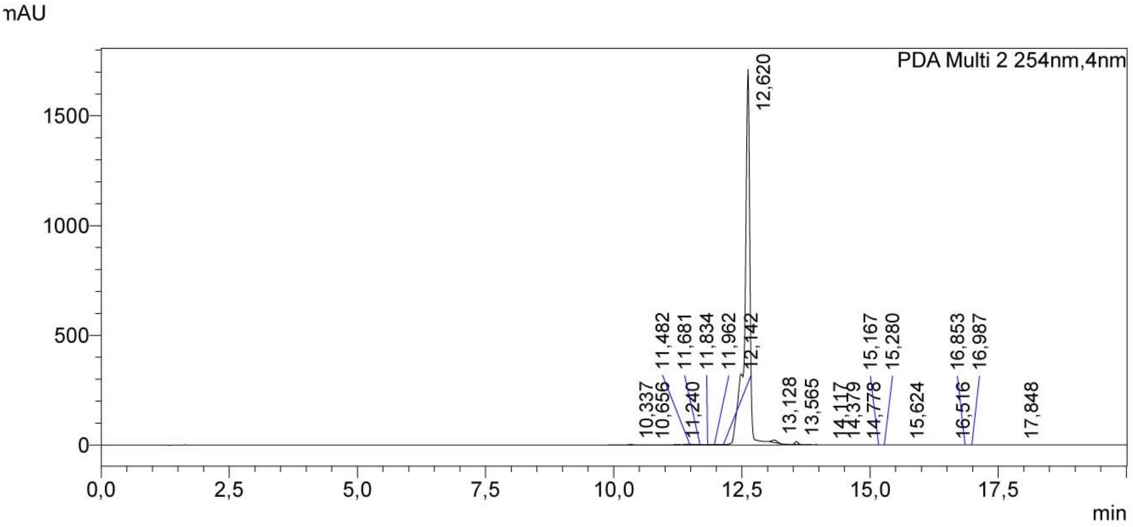

PDA Ch2 254nm

Peak#	Ret. Time	Area	Area%	Height
1	10,337	48668	0,371	3046
2	10,656	11140	0,085	598
3	11,240	36404	0,278	1762
4	11,482	43210	0,329	3163
5	11,681	13416	0,102	1717
6	11,834	13402	0,102	1814
7	11,962	11248	0,086	1507
8	12,142	16974	0,129	2347
9	12,620	12765720	97,333	1711211
10	13,128	74065	0,565	10751
11	13,565	62125	0,474	14355
12	14,117	1314	0,010	176
13	14,379	1063	0,008	148
14	14,778	1594	0,012	216
15	15,167	2154	0,016	365
16	15,280	2384	0,018	309
17	15,624	3452	0,026	481
18	16,516	2248	0,017	237
19	16,853	1105	0,008	159
20	16,987	1747	0,013	222

Peak#	Ret. Time	Area	Area%	Height
21	17,848	2028	0,015	204
Total		13115461	100,000	1754789

Figure S4. HPLC chromatogram of compound **11**.

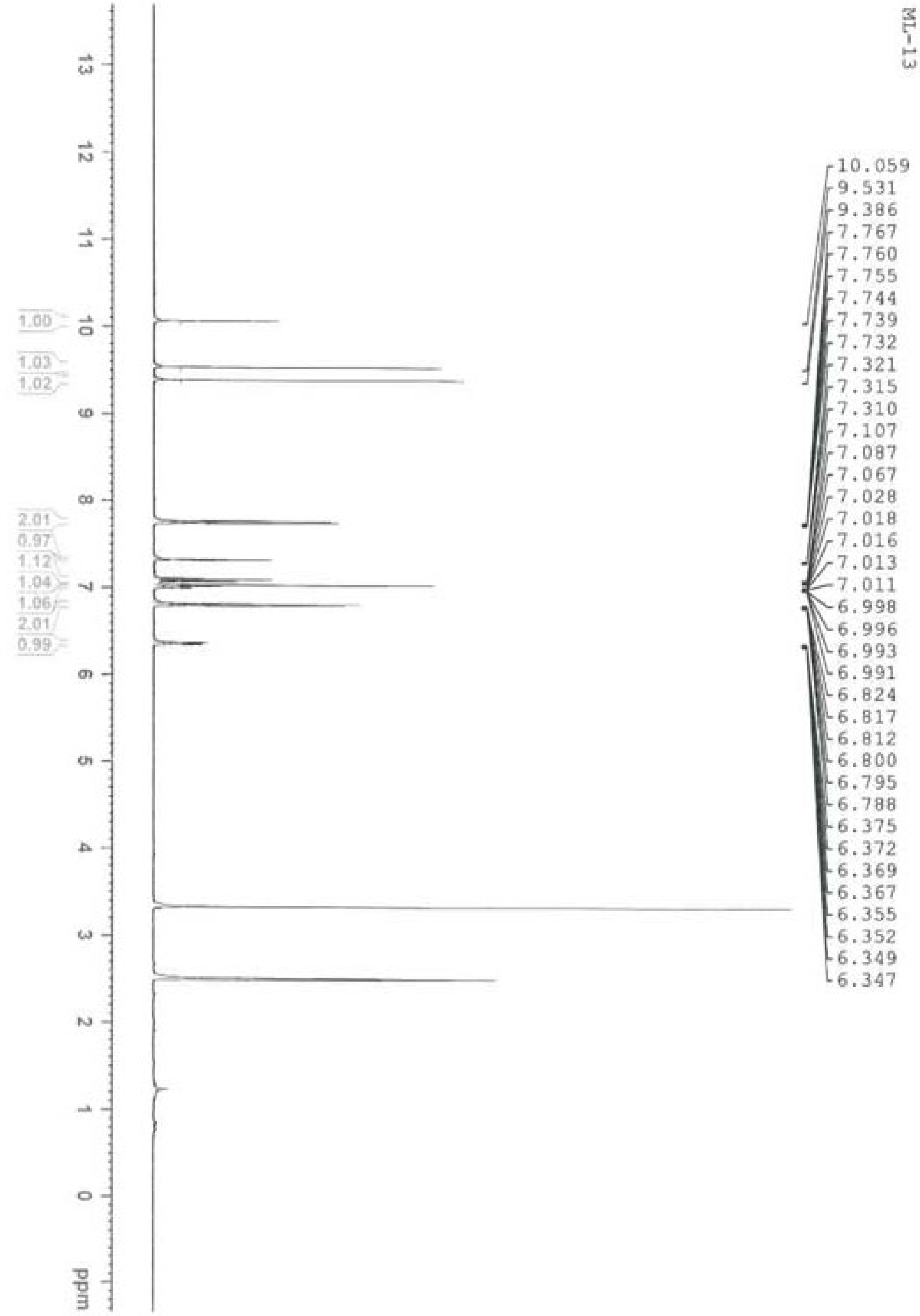

Figure S5. ¹H-NMR of compound **8**.

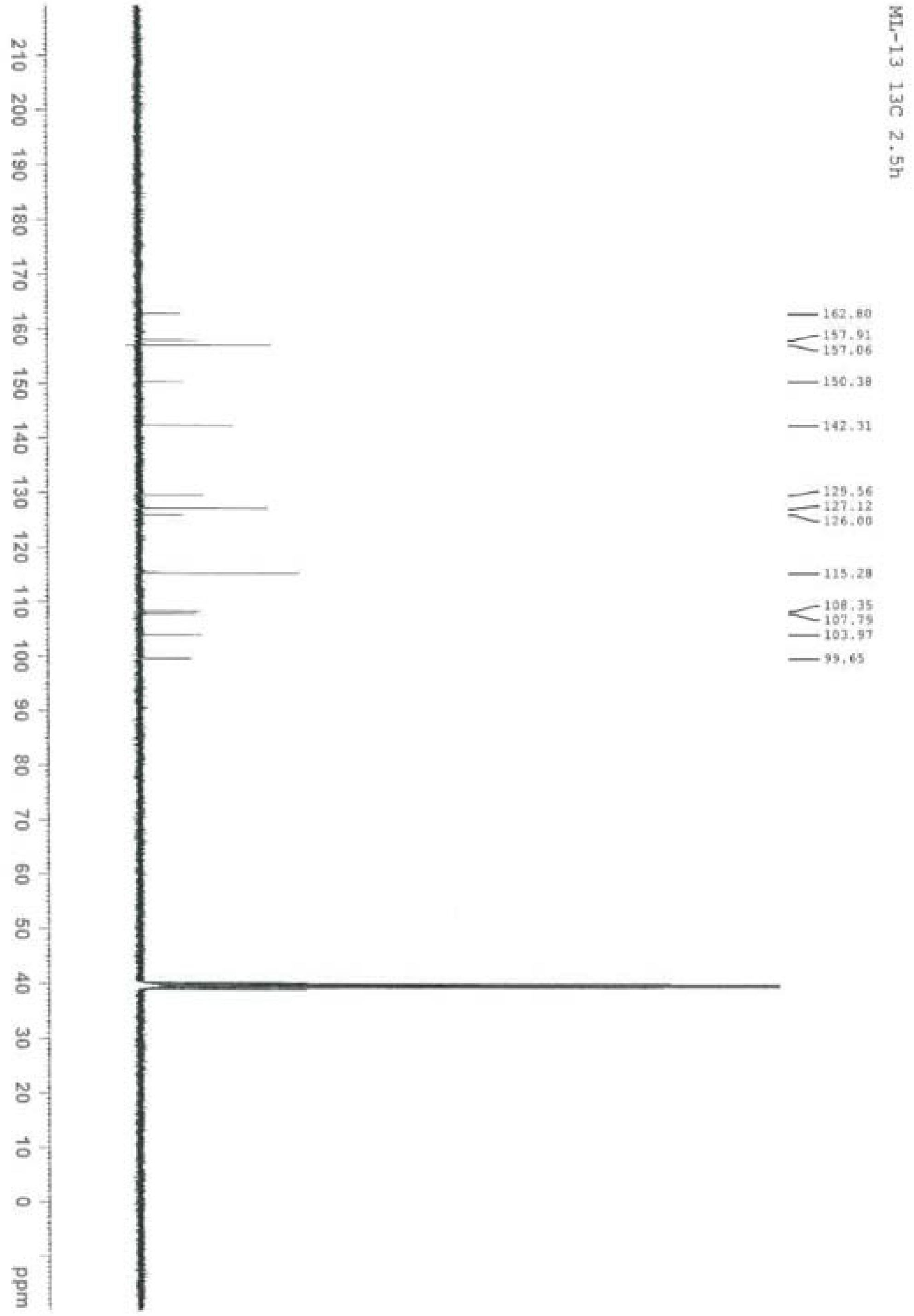

Figure S6. ^{13}C-NMR of compound **8**.

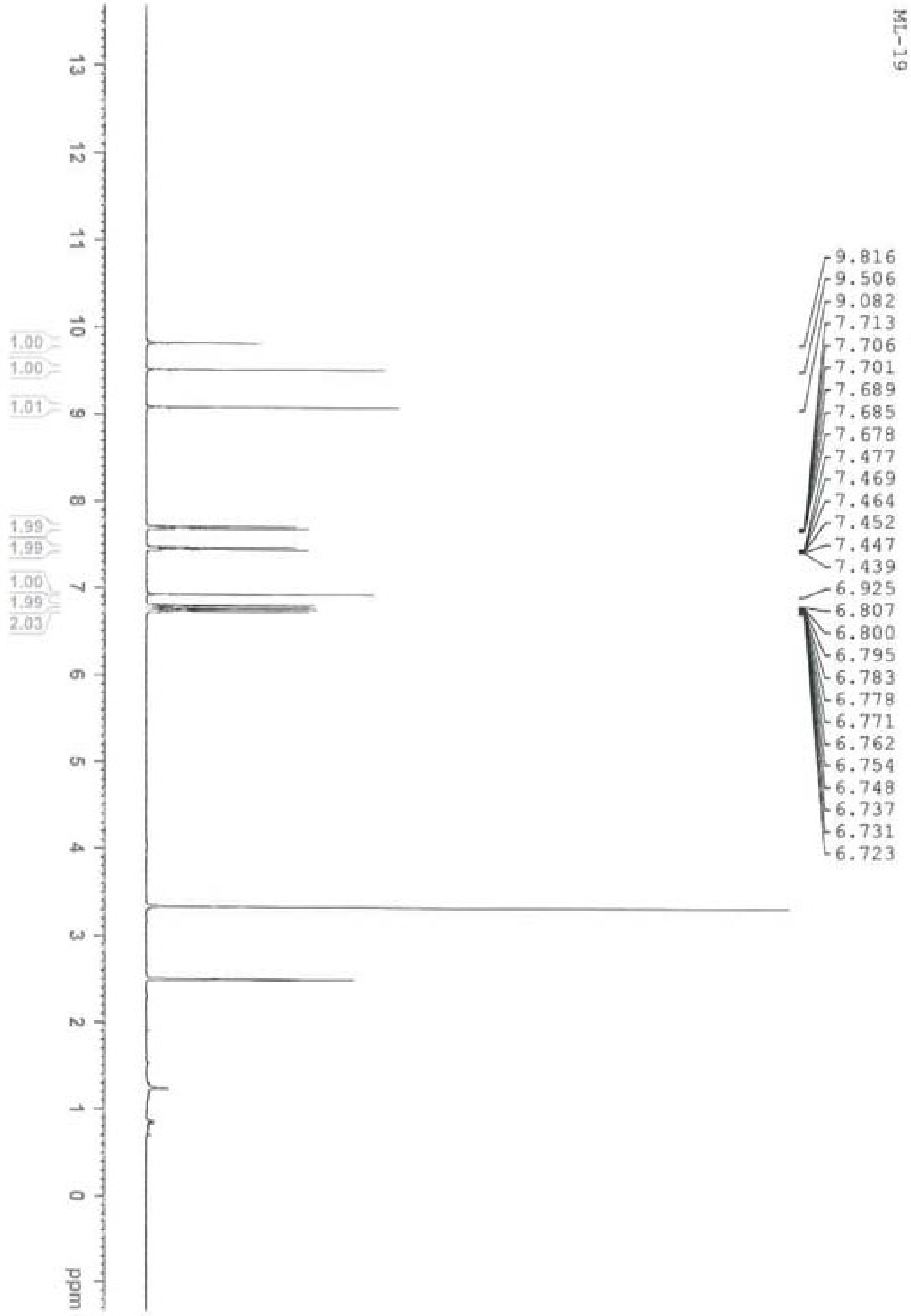

Figure S7. ^{1}H-NMR of compound **9**.

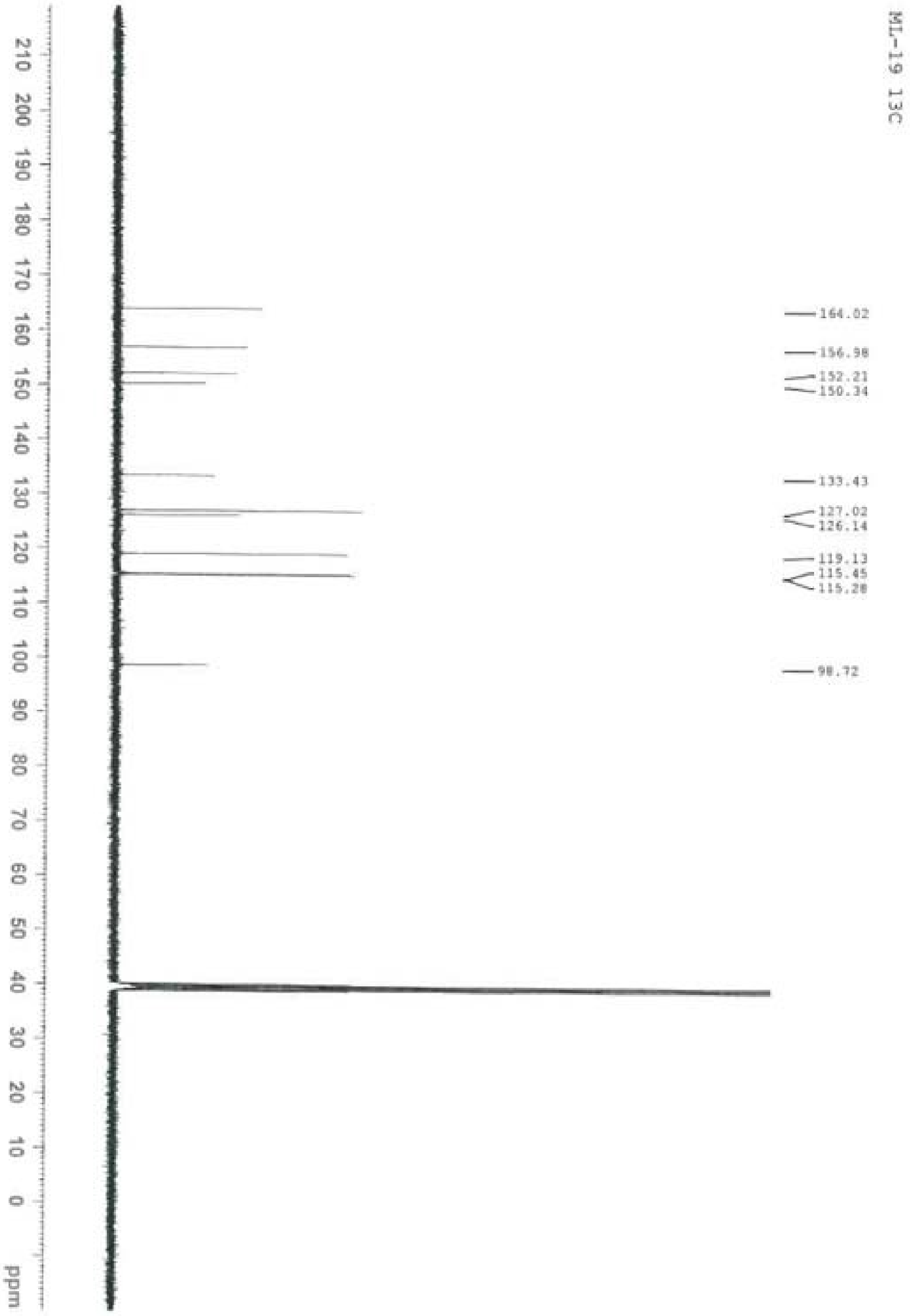

Figure S8. ^{13}C-NMR of compound **9**.

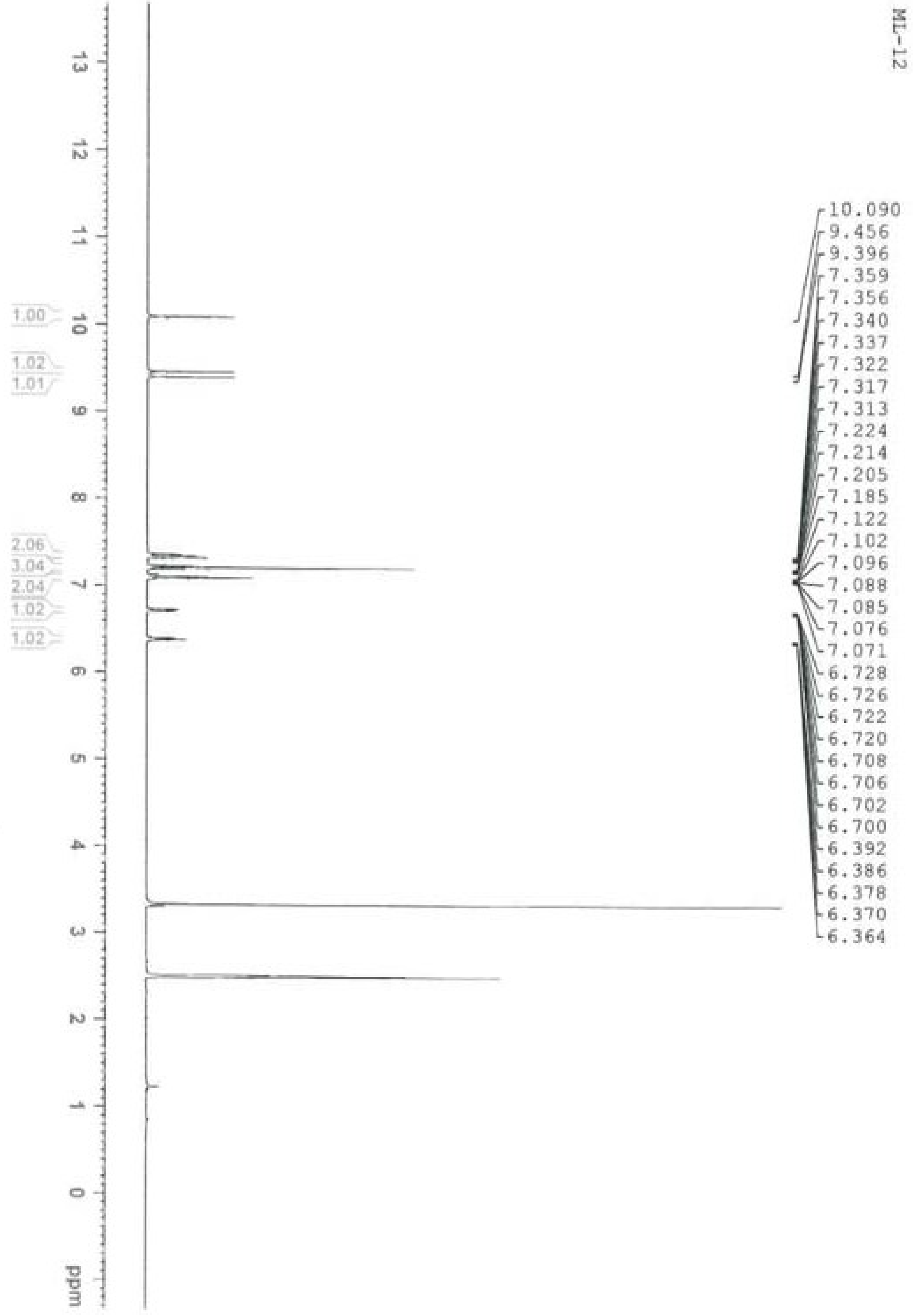

Figure S9. ^{1}H-NMR of compound **10**.

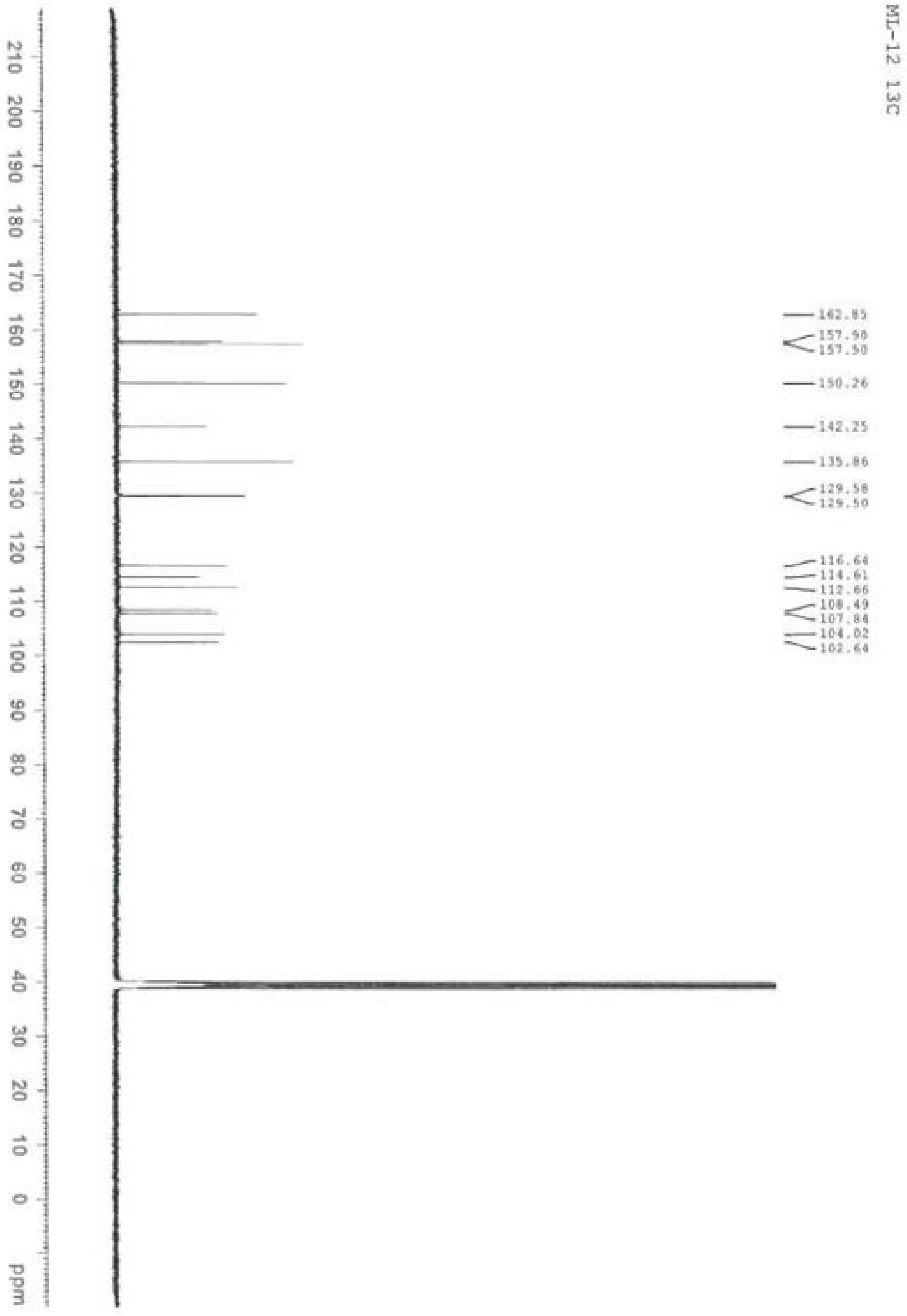

Figure S10. ^{13}C-NMR of compound **10**.

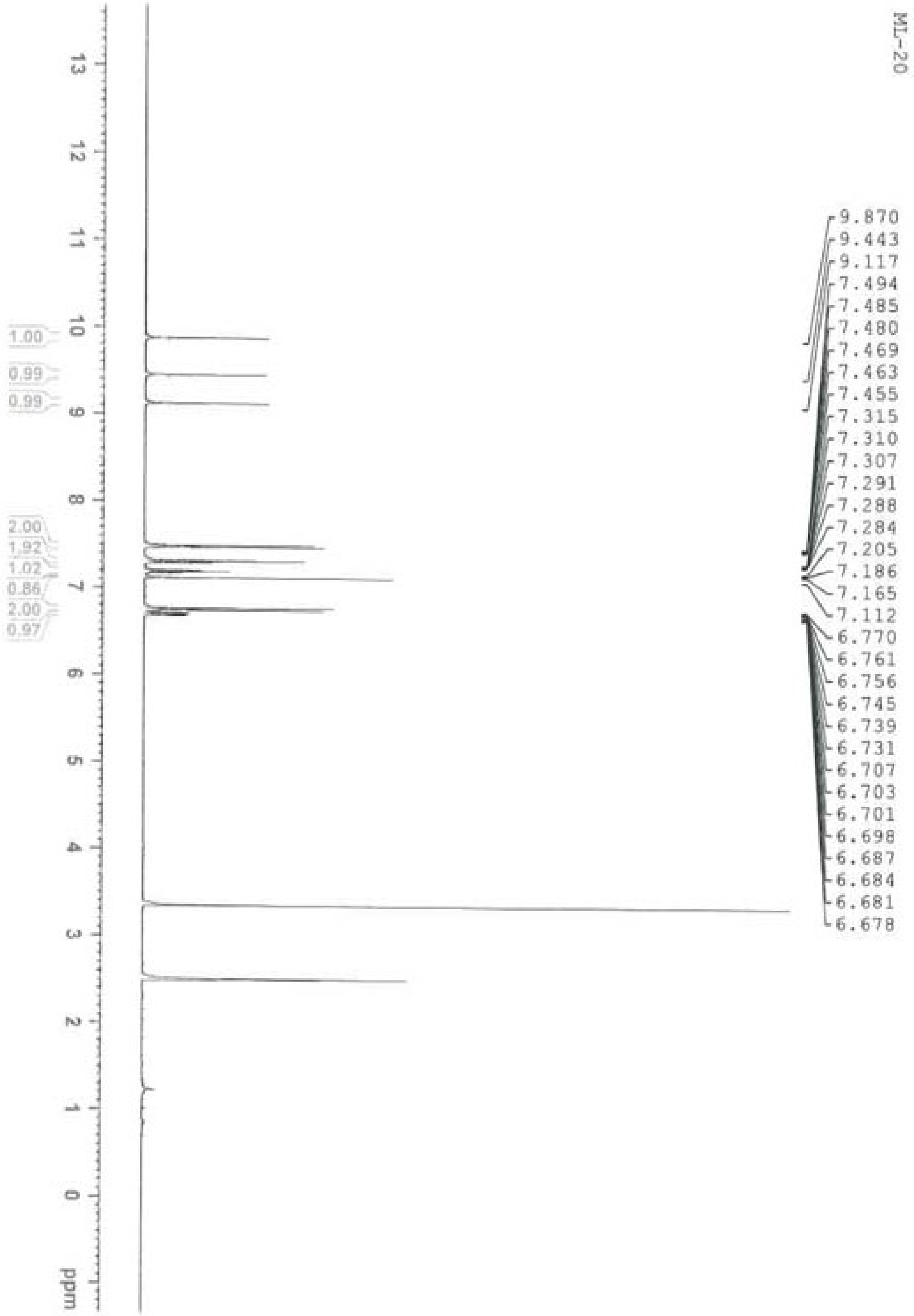

Figure S11. ^{1}H-NMR of compound **11**.

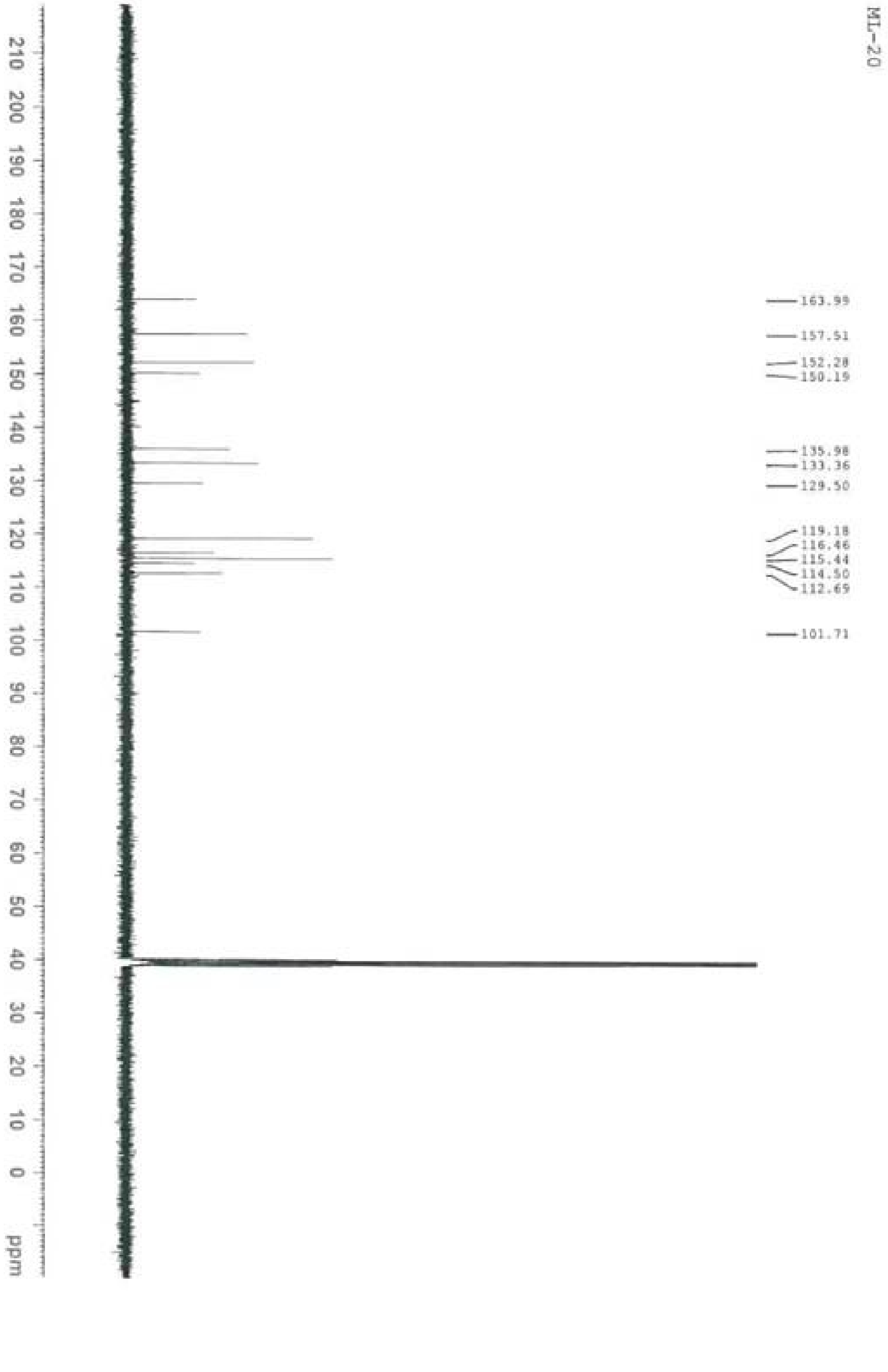

Figure S12. ^{13}C-NMR of compound **11**.

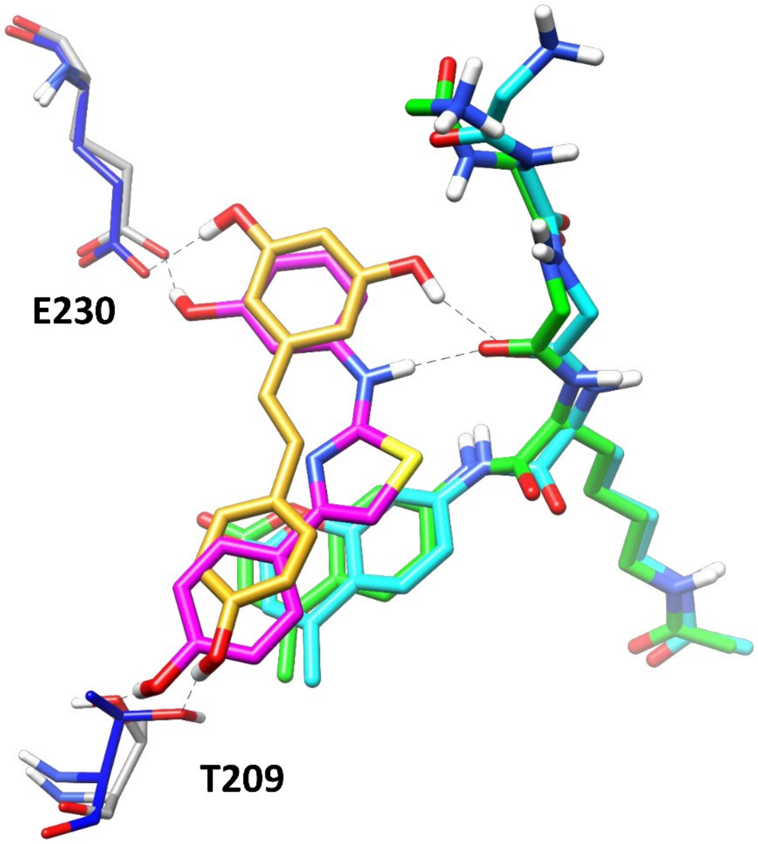

Figure S13. Minimized average structure of SIRT1/p53-AMC/**8** complex superimposed to the MD-refined SIRT1/p53-AMC/resveratrol complex. In the **8**-bound complex, ligand, peptide and protein residues are shown in purple, green and grey, respectively, while in the resveratrol-bound complex they are shown in gold, cyan and blue, respectively. For clarity only the ligand molecules occupying site 1 and the two protein residues (T209 and E230) forming H-bonds with them are shown. For the same reason, the side chains of R1, H2 and K3 of the p53-AMC peptide are not displayed.

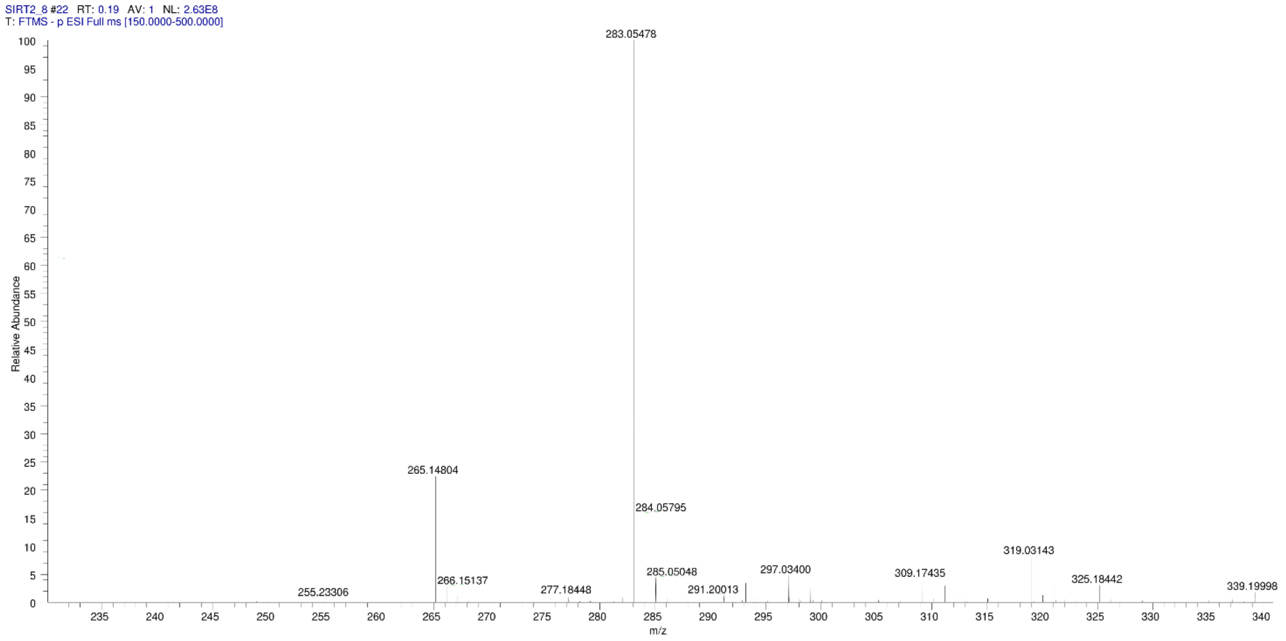

Figure S14. ESI-HRMS spectrum of compound **8**.

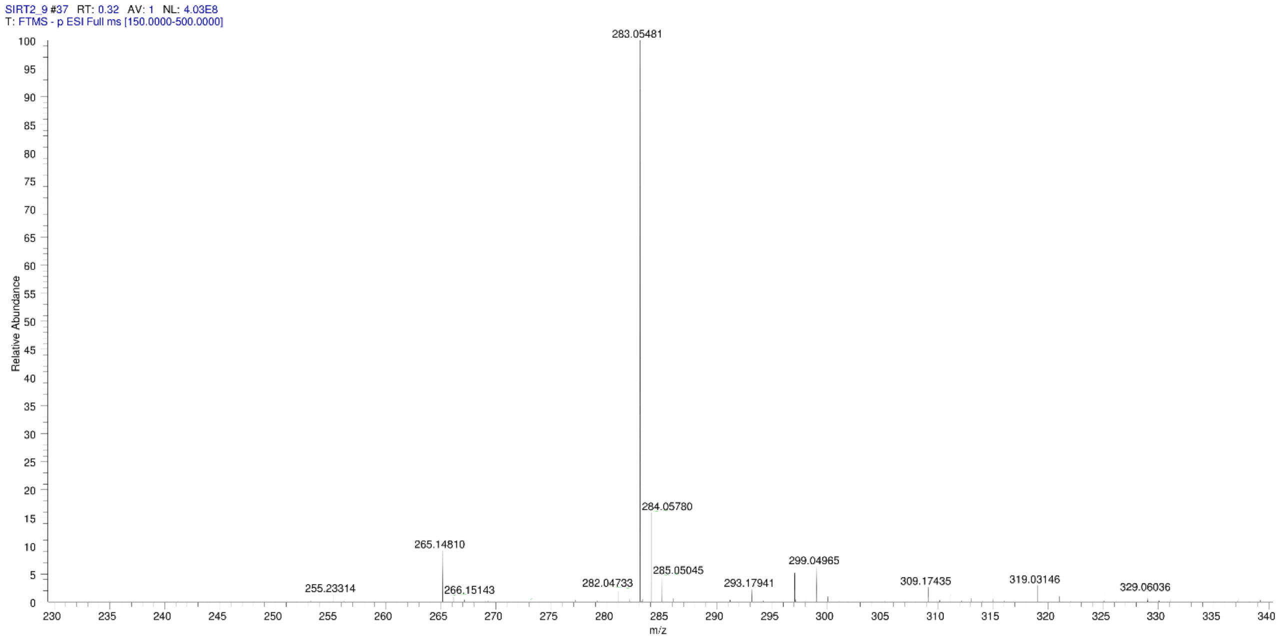

Figure S15. ESI-HRMS spectrum of compound **9**.

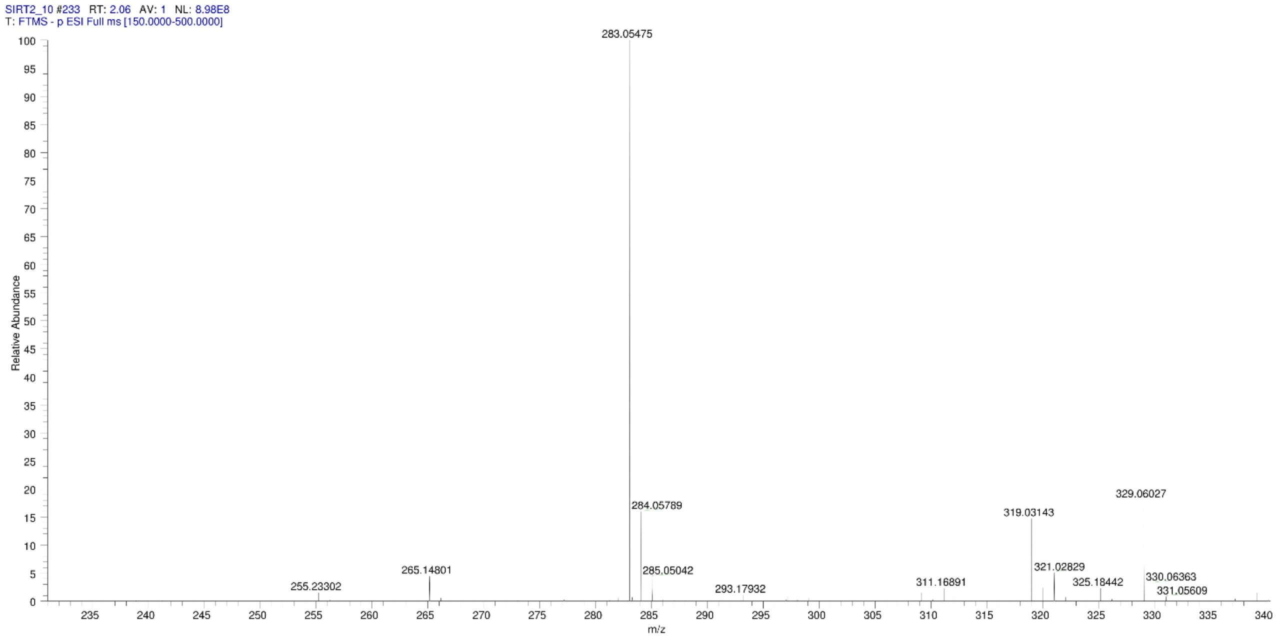

Figure S16. ESI-HRMS spectrum of compound **10**.

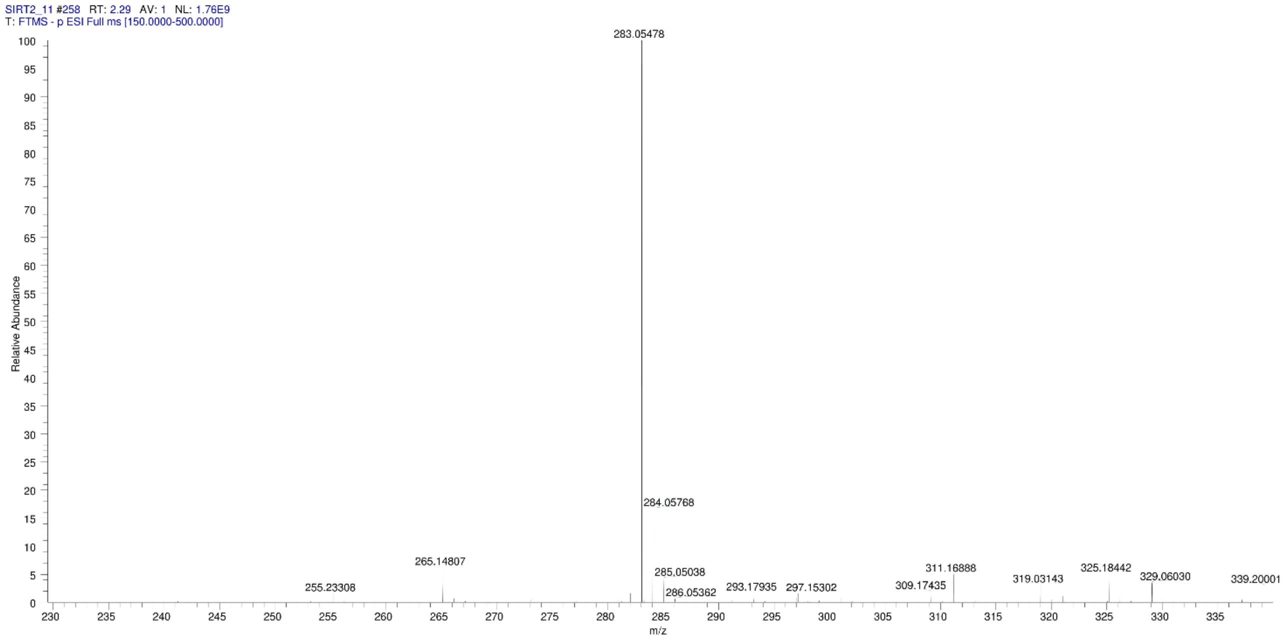

Figure S17. ESI-HRMS spectrum of compound **11**.
